# Knowledge, Attitudes and Practices of the Lebanese Community toward Food Adulteration

**DOI:** 10.3390/foods11203178

**Published:** 2022-10-12

**Authors:** May Khanafer, Marwa Diab El Harake, Imad Toufeili, Samer A. Kharroubi

**Affiliations:** Department of Nutrition and Food Sciences, Faculty of Agricultural and Food Sciences, American University of Beirut, Riad El Solh, P.O. Box 11-0236, Beirut 1107-2020, Lebanon

**Keywords:** food adulteration, knowledge, attitudes, buying practices, Lebanon

## Abstract

Food adulteration is the purposeful act of decreasing the quality of food goods offered for sale, whether by adding or replacing inferior substances or by the removal of some valuable ingredient. A limited number of studies have explored the knowledge, attitudes and practices (KAPs) concerning food adulteration in Lebanon. The objectives of the present study were to determine the knowledge, attitudes and practices of identifying adulteration in the process of food purchase by Lebanese adult consumers, and to identify factors associated with food adulteration. An online survey (*n* = 499) was administered among Lebanese adults aged 18 years and above. Results showed that the majority had a low food adulteration knowledge score (73.1%). During shopping, fewer than half of the participants checked the ingredients (42%) and nutrition facts label (33.9%). Regression analyses showed that six predictors were significantly associated with participants’ knowledge scores including gender, age, marital status, education (undergraduate and master degree) and employment status (student). The results of this study show that knowledge and practices of identifying adulteration in the process of food purchase by consumers are lacking among most respondents. Increasing knowledge, awareness and motivation to identify food adulteration products during food shopping will empower consumers to improve buying practices, especially for the public with a lower level of education.

## 1. Introduction

Food adulteration is referred to as a “silent genocide” and, according to the UK Food Standard Agency (FSA), it is defined as “deliberately placing food on the market, for financial gain, with the intention of deceiving the consumer” [1]. Economically, the food manufacturers receive an increase in their profits. However, food adulteration is associated with serious health outcomes varying from minor sicknesses such as gastrointestinal diseases (e.g., vomiting and diarrhea) to major diseases such as cancer or even death.

Food adulteration can be performed in various ways. The first way is by adding extraneous matter, such as adding chalk to powdered milk or adding lead to turmeric. The second is by mixing inferior quality with superior quality; this is commonly found in honey, which is mixed with sugar syrup. The third is adding illegal preservatives and coloring dyes, such as coloring wine or spices, and the fourth is when companies remove vital ingredients, such as replacing milk fat with vegetable fat. The Administration Assistance and Cooperation System for Food Fraud (AACFF), created to communicate information on non-compliance with and potential intentional violations of the EU agri-food chain legislation, classified the methods of food adulteration into four more different ways, which include mislabeling; the replacement/dilution/removal of products, unapproved treatments or processes; absent/falsified or manipulated documents; and finally, Intellectual Property Rights (IPR) infringement [2]. On top of the various ways of adulterating food, there are also different types of adulterants. First are intentional adulterants such as chalk powder, mud, pebbles and others. The second is metallic contamination, which is lead, mercury, tin and so on. The final type is incidental adulterants, which are pesticide residues, dropping of rodents or larvae on foods [3].

The EU Food Fraud Network took part in Operation OPSON, which is a joint Interpol/Europol initiative where they target food adulteration. Sixteen EU member states and 18 non-EU member states (including UAE and Egypt) joined in this initiative. One of the missions was on the adulteration of organic foods where most claimed to be organic even though they were not (mislabeling). Over 775 tons of adulterated/counterfeit organic foods were detected in 2019. Another was on the adulteration of pure Arabica coffee where the Arabica coffee was found to be replaced with a cheaper bean called Robusta (replacement of product). Sixteen EU countries took part in this investigation and the Germans led it. It was found that out of 400 coffee samples, 10 of them were adulterated, which caused the launching of further and deeper investigations [2].

A review of 44 recent studies from many different countries, including the UK, the US and Asia, analyzing more than 9000 seafood samples from different sources/food items revealed that 36% were mislabeled and, shockingly, some samples proved not to be entire aquatic species. In Singapore, for example, prawn balls were found to contain pork instead of prawn [4]. In Bangladesh, 73% of the juices in Dhaka City failed to conform to the standards set by the Bangladesh Standards and Testing Institute, with most of them having incorrect production and expiration dates [5]. Furthermore, out of 62 salt samples, 87% of them did not conform to the standard levels of pH, iodine, chlorine and moisture levels [5].

Knowledge is essential for the consumer to protect themselves and their families against faulty buying practices. In Karnataka, India, it was found that only 21% of study participants had good food adulteration knowledge, mainly among those with a higher education and of a younger age [1]. Similarly, in India, 73% of cloves and 61% of tea in Wardha District were found to be adulterated and the purity of food was highest among “literate” participants and lowest among the “illiterate” participants [6]. Additionally, some socioeconomic factors were found to be associated with good practices, such as checking the nutrition label, FSSAI logo and expiry date, including age, education status and socioeconomic status.

Furthermore, China has faced many food adulteration incidents that have undermined its integrity and led to an increased anxiety and reduced trust regarding the authenticity, safety and quality of food that is available [7]. One example is the famous milk scandal in China in 2008 involving the adulteration of infant formula and powdered milk with the poisonous melamine, which caused over 6240 cases of kidney stones in children and three deaths [8]. In total, 47 countries received melamine-contaminated products, as reported to INFOSAN or published on each country’s official government website, either through direct import or through third countries [8]. Research proved that despite significant reforms to food safety governance in China implemented by respective food safety acts (The Food Safety Law 2009 and 2015), Chinese consumer trust in the domestic food system remains low [7]. Notably, the addition of melamine was also found in 2008 in Poland in a wheat-based snack called Salty Sticks [9].

In Lebanon, a limited number of studies have explored the prevalence of food adulteration. Honey is often a target for adulteration through the addition of different sugar syrups during or after honey production, resulting in a reduction in its nutritive value [10]. Out of 33 Lebanese honey samples analyzed, 20 samples were classified as authentic and 13 as adulterated honey. Furthermore, since carob molasses as a product is sold under the claim that it has no added sugar and is free of any foreign material, there is a need to develop easy to conduct and low-cost methods as tools to detect these adulterations. Another study showed high levels of adulterations of carob molasses in the Lebanese market. In addition, polycyclic aromatic hydrocarbons (PAHs) were detected in 60% of olive oil brands (41% of samples) where 12% of brands contained traces of probably carcinogenic (Class 2A) compounds and 56% of brands contained traces of possibly carcinogenic (Class 2B) compounds [11]. Additionally, the lower quality of this brand (KHA) was evident during analysis of the oil, where acetonitrile used in the elution appeared to dissolve colorants that adulterated the olive oil [12]. Another Lebanese study evaluated the gluten contamination in 173 (gluten free) labeled food products and found that 19% of the total samples were found to be mislabeled [13]. On 22 July 2020, the Minister of Public Health discovered that the Lebanese Poultry Company were supplying supermarkets and restaurants with expired products, some dating back 4 years, and disguising them as chicken nuggets and burger patties as well as changing their expiration dates.

In 2017, the knowledge and behaviors of the Lebanese population toward the adulteration of honey were studied, and it was found that around 50% of the participants checked if the honey was adulterated and 75% claimed to know a couple of adulteration methods [14]. Notably, local honey in Lebanon is priced higher than that imported, and 91% of Lebanese participants were not attracted to the low-priced honey; they believed that if the honey is placed at a high price, then it is “safe”. However, imported honey goes through several ISO testing processes, whereas the local honey is not tested [14].

Back in 2011, the Agriculture Ministry and Consumers in Lebanon reported that there were several olive oil brands on the market containing toxic chemicals [15]. Confirmed by the head of Consumers Lebanon, Zuheir Berro, reports showed a lack of monitoring in Lebanese law that caused the fraudulent olive oil issue in the market. The head of Consumers Lebanon said several olive oil producers in Lebanon were adding certain chemical ingredients such as benzopyrene and acrolein during the extraction stage of olive oil production, which is not healthy for human health. The statement made by the National Committee for Oils and Fats (NCOF) in Lebanon noted that the risks of the toxic chemicals used in the tainted olive oils could increase the chances of cancer. NCOF warned the public by stating that “the brains of children may be permanently harmed from consuming such oil products because fats present in such oils will, in the long run, substitute the healthy fats of the brain” [15].

Unfortunately, Lebanon does not carry out enough tests and preventative measures for the beverages and foods we consume, and we also do not have enough awareness regarding this topic. Furthermore, a limited number of studies have explored the knowledge, attitudes and practices (KAPs) concerning food adulteration in Lebanon. Therefore, the aim of this study is to assess the level of knowledge, attitudes and practices of identifying adulteration in the process of food purchase by Lebanese consumers. The main research objectives include (i) to investigate the level of knowledge, attitudes and practices related to identifying food adulteration while food shopping among Lebanese consumers, (ii) to identify factors that would affect consumers’ knowledge and behaviors and (iii) to provide recommendations to address these gaps/challenges as well as opportunities to improve consumer behavior related to food purchasing in Lebanon.

## 2. Materials and Methods

### 2.1. Study Design and Sampling

This descriptive study was based on an online survey conducted between January and February 2021 among a sample of Lebanese citizens or residents of Lebanon aged from 18 to 71 years of age. Sample size calculations showed that a minimum of 577 participants ought to be recruited to estimate a prevalence of 50% with a 95% CI and a margin of error of 5% and a design effect of 1.5. The sample size was calculated using the WHO sample size calculator available at: www.who.int/ncds/surveillance/steps/resources/sample_size_calculator.xls, accessed on 11 December 2019 [16].

### 2.2. Data Collection

The research team was CITI certified and received training on conducting research with human subjects according to AUB IRB regulations prior to the initiation of the study. An online invitation was shared and posted via different social media platforms of the graduate students (WhatsApp groups, Facebook pages, Instagram, and Twitter). Before starting the survey, participants were asked to complete a consent form, which appeared on their screen (see Supplementary S1 in the Supplementary Materials). The completion of the survey took approximately 5–10 min (Supplementary S2; Supplementary Materials). Participants’ identity was completely anonymous and participation in the survey was voluntary.

### 2.3. Survey Format

The survey was developed to evaluate the knowledge, attitudes and practices toward food adulteration. The survey was based on previous similar studies [3,5,10] and composed of three sections. The first section included questions related to participants’ sociodemographic characteristics such as age, gender, relationship status, area of residency, educational level, employment status and the household monthly income. The second section was composed of 7 questions related to the participants’ buying/shopping and consumption practices. For example, participants were asked: “Who buys the groceries and what do you look out for before buying or consuming a product?”. Additionally, this section included questions related to participants’ attitudes toward labeled and branded products. Notably, branded products are all industrial products that are found in the supermarket; however, unbranded products are non-industrial products where most do not even include nutritional facts and are bought from farms and households. The last section included 9 questions to assess participants’ knowledge on food adulteration. It comprised of 4 multiple choice and 2 yes/no questions about food adulteration, and 3 questions relating to their opinions on the Lebanese law and its reinforcement. The complete survey is available in Supplementary S3 of the Supplementary Materials.

### 2.4. Data Assessment

A month after initiating the online survey, data were collected from January till February 2021. Data were extracted, cleaned, entered and statistically analyzed using the Statistical Package for the Social Sciences (SPSS) version 25.0 (SPSS Inc., Chicago, IL, USA). Out of 561 participants who participated in the survey, we had 499 participants with complete responses (89% response rate) and 62 participants were excluded from the study for incomplete responses. A knowledge score was created by summing up the number of correct answers for each participant. Participants’ responses for the following questions were included in the computed scores: “How is food adulterated?” (0–6 points), where choosing “rotten bread” would be the wrong answer; “Which substance(s) do you believe can be considered adulterants?” (0–6 points); and “Which foods do you believe can be adulterated?” (0–8 points), where choosing all the options would have been correct; each correct response was 1 point. Then, participants’ total response score was calculated, ranging from 0 to 20 points. Participants’ knowledge scores were then used to classify participants with low score (0–10) and high score (11–20).

Descriptive statistics were presented as means and standard deviations (SD) for continuous variables and as frequencies and proportions for the categorical variables. The food adulteration knowledge score was considered as a continuous variable, with no specific cut-off, whereby higher values indicated better knowledge. Simple and multiple linear regression analyses were used to investigate the associations of sociodemographic factors with knowledge, using the knowledge score as dependent variable and the sociodemographic factors as independent variables. Results from the linear regression models were expressed as beta coefficients (β) with 95% confidence intervals (CI). All reported *p*-values were based on two-sided tests and were compared with a significance level of 5%.

## 3. Results

### 3.1. Sociodemographic Characteristics of Participants

All the sociodemographic characteristics of the study population are presented in Table 1. More than half of the study participants were males (59.7%) and ranged between 18 and 71 years of age with an overall mean (SD) age of 26.4 (8.7) years. The participants were of varied age groups, with most of them aged below 40 years (*n* = 464, 93%), and the majority were single (*n* = 415, 83.2%). As for participants’ highest education level, 14.8% had a high school diploma, 52.1% had an undergraduate degree, 29.5% had a master’s degree and 3.6% had a postgraduate degree. Most of the participants resided in Mount Lebanon (*n* = 303, 60.7%) and Beirut (*n* = 142, 28.5%) and the remaining were in Beqaa (3.8%), North (4.0%) and South (3.0%). Regarding employment, approximately 43.1% of participants had a full-time job, 12% had a part-time job, 28.7% were studying and 15.6% were either seeking employment or unemployed. More than a third of participants (39.7%) had a total monthly income of more than LBP 5,000,000, 28.3% had a monthly income between LBP 1,000,000–3,000,000, 20.4% had a monthly income between LBP 3,000,0000–5,000,000 and a small minority had a monthly income below LBP 1,000,000 (9.2%).

### 3.2. Knowledge on Food Adulteration

The food adulteration knowledge of Lebanese consumers is shown in Table 2. More than three quarters of participants (77.0%) were knowledgeable that food adulteration can affect the health of individuals. When asked how food can be adulterated, more than half of study participants correctly chose “changing expiry date” (58.3%), followed by “coloring dyes added to tea” (47.9%), “water added to milk bottle” (46.5%), “chalk added to turmeric” (38.9%) and “claiming milk is lactose free although it is not” (34.7%). However, the majority of participants chose the wrong option “rotten bread” (71.1%). Participants’ responses to food adulterants included: “illegal colorants and preserves” (52.5%), “Chalk” (32.1%), “Water” (27.5%), “Sand” (19.4%), “urea” (14.2%) and “pebbles” (14.2%). Concerning the foods that can be adulterated, participants chose chicken, meats and meat products (59.5%), milk (52.3%), wheat, juice (51.1%), flour and bakery products (50.5%) and spices (34.9%).

With regards to the knowledge score, the majority of participants had low food adulteration knowledge (73.1%) with a mean score of 7.74 (Table 2).

In Table 3, simple linear regression shows that six predictors were significantly associated with participants’ knowledge scores, including gender (*β* = −0.900, *p* = 0.031), age (*β* = 1.368, *p* = 0.048), marital status (*β* = 1.096, *p* = 0.041), education (undergraduate degree (*β* = 1.376, *p* = 0.020), master’s degree (*β* = 1.256, *p* = 0.049)) and employment status of students (*β* = −1.061, *p* = 0.028). Multiple regression analyses (Table 3) also showed that employment remained significantly associated with knowledge score. Being a student significantly decreases the food adulteration knowledge score by 1.051 points (*β* = −1.051, *p* = 0.038).

### 3.3. Attitudes toward Food Products

When asked whether the consumer buys mainly branded or unbranded products, 81% of Lebanese consumers chose branded products and 18.4% preferred unbranded products (Table 4). The most common unbranded items were found to be olive oil (65.1%) and honey (58.7%). On the other hand, participants specified buying unbranded rice (16%) and molasses (29.5%). Regarding attitudes toward food labels, 38.9% of participants mainly indicated that they completely trust the labels, and 36.7% of participants mentioned that they only trust labels on imported brands. Nearly a quarter of participants did not trust the labels at all (22%) and fewer than 5% of participants trusted local brands (2.4%). Regarding the Lebanese laws, 66.9% of the participants believed that Lebanon does not have a law against food adulteration, with only 33.1% believing that there is a law. Nonetheless, only 2.4% believed that the law is being followed. Moreover, fewer than half of the study participants believed that the rate of food fraud in Lebanon is high (45.9%).

### 3.4. Buying Practices

In Table 5, the results show that 62.3% of the participants read the food labels before buying or consuming any food product. Those who usually read food labels before shopping any product were then asked to choose what they read on the label from a list of items. Participants read food labels to check for several aspects: including the ingredients (42%), the nutrition facts label (33.9%), the nutrition claims (20.2%), just the calories (15.2%), the storage instructions (14.6%), the addition of food additives (13.0%) and health claims (12.0%). Moreover, most participants indicated that when buying any product, they mainly focused/looked at the expiry date (81.8%), the price of the product (76.6%) and the brand (68.9%).

## 4. Discussion

To the best of our knowledge, this study is the first to evaluate the food adulteration knowledge, attitudes and practices in Lebanon. The results showed that there are gaps in the knowledge among the study population. Additionally, significant associations were found between sociodemographic characteristics and knowledge. We grasped a good idea on the buying practices as well as the attitudes toward branded and unbranded products and the level of trust in them.

In our study, 37.7% of the study participants claimed that they do not read the labels before buying or consuming any product. Similarly, in a study conducted in 2017 to examine the usage and understanding of food labels in Lebanon, the authors concluded that the most common reasons for not reading the labels were: 34.9% did not have enough time to, 15.1% believed that there is no need to, 9.8% had no knowledge on how to read them and 8.0% found the labels too small [17].

Buying branded products does not necessarily mean that the product will be completely fraud free; however, since most companies perform tests obliged by the government, the risks of food adulteration decrease. In Lebanon, buying unbranded products from the village is common in almost every household. Only 18.4% claimed that they never buy any unbranded product and the remainder always have at least one of the following unbranded products in their kitchen: honey, olive oil, molasses and rice. Unfortunately, Lebanon does not have strict rules obliging all food products that are sold to be tested. The participants reported a lack of trust in the government’s ability to suspect food adulteration and apply laws, with only 2.4% believing that the law is being followed. This is similar to the Chinese where trust in the domestic food system remains low [7]. Nonetheless, the participants’ trust for food labels, in general, is below average, where 38.9% “trust the labels completely” and 36.7% “only trust labels on imported brands”. However, it has been shown in several studies that mislabeling is popular all over the world. The Administration Assistance and Cooperation System for Food Fraud (AACFF) calculated how many times each method of adulteration was caught in 2019 and concluded that the most popular was mislabeling (47%). The replacement, dilution, addition or removal in products was 20%, unapproved processes were 16%, document manipulation, falsification or absence was 15% and IPR infringement was 2% [2]. Even though 58% of the participants knew that “changing the expiration date” is considered as food adulteration, the lack of knowledge toward adulteration via mislabeling was shown by “claiming milk is lactose free although it is not” being chosen the least number of times as a method of adulteration (34.7%).

When asked about substances that can be adulterants, our results showed that participants correctly selected colorants and preservatives. However, the majority of participants chose the one wrong option “rotten bread”. It is important to note that rotten bread is not an adulterant since naturally, over time, bread will rot. Rotten bread is a food safety risk but not considered food adulteration. Similarly, a study conducted on residents in Dhaka City, Bangladesh, found that the most common response was “rotten bread” and the most used adulterants were “colorants” [5]. However, the Lebanese study showed that the participants chose “water” 137 times (27.5%), whereas in Bangladesh it was chosen only once (1%). On the other hand, “urea” was chosen 71 times (14.2%) in Lebanon but 22 times (23%) in Bangladesh [5]. Even though the percentage in Bangladesh is lower, it is important to note that their sample consisted of 50 participants instead of 499. One common approach for adulterating milk is to mix water in it and then add urea to the resultant milk to raise its “solid not fat (SNF) value” and give it a concentrated and rich appearance [18]. Even though urea is a natural end-product of nitrogen metabolism and a normal constituent of milk, a limit concentration in milk is normally accepted to be less than 70 mg/dl [19].

As for total knowledge scores, among 499 participants, 73.1% had a low knowledge score and 26.9% had a high knowledge score. It should be noticed that even though 384 people (77.0%) chose that food adulteration can harm our health, 101 of them (20.2%) did not know whether or not it does and 14 claimed it does not (2.8%). Additionally, the participants with undergraduate degrees had higher scores than those with a high school diploma, whereas those with master’s degree had higher scores than both. People aged 30–39 had better knowledge than those aged 18–29. The participants who were students had lower scores than the ones who were employed. The relationship between knowledge score and employment also proved to be statistically significant in the multiple regression, proving that students had lower scores than employed participants. This factor is most probably related to the level of education reached. Comparing these results to those of another similar study conducted in Karnataka, India, they found that among 75 participants, only 21% had good knowledge, 60% had average knowledge and the remaining 19% had no knowledge [20]. As with this study, they found a statistically significant association between knowledge of adulteration and educational level: the higher the education, the more they are aware of adulteration. Furthermore, they found a statistically significant association between knowledge of adulteration and age, where people aged 25–50 years old had a better knowledge than those over the age of 50 [18]. A previous study also showed that the food hygiene knowledge score was significantly affected by gender, age, marital status, educational level and the major of study.

In Lebanon, the law against adulteration by the Lebanese parliament under the Consumer Protection Law Article 10 states that those involved: “Shall be punished by imprisonment from three months to one year and by a fine varying from LBP 25 to 50 million, whoever knowingly commits the following acts:

- To adulterate ingredients of human and animal foods, or pharmaceuticals products, or drinks or industrial, agricultural or natural products.
- To trade in or circulate spoiled, polluted or expired foodstuffs.
- To possess products or foodstuffs of the kind prescribed in the above clauses (Article 10, 2004)”.

In our study, it was found that 66.9% of the participants believed that Lebanon does not have a law against food adulteration, with only 33.1% believing that there is a law. Nonetheless, only 2.4% believed that the law is being followed. Moreover, fewer than half of the study participants believed that the rate of food fraud in Lebanon is high (45.9%). The law protecting the Lebanese population is available; however, not enough testing is happening on imported and local products to find these adulterated foods. The government must conduct routine testing with all food categories and penalize companies who do not conform to the basic food standards. Awareness is key to decreasing this problem as well; by talking about it via social media or on the news not only gives people the information needed but also warns food and beverage companies to not disobey the law.

Despite its limitations related to the design, given that the sampling was not a true random sample, in addition to the fact that the data consisted of self-reports and were collected using online surveys, although many people do not have access to the internet or do not have smart phones to fill them in, and that participants were not supervised, hence they had the chance to search online and give us the correct answers, introducing information bias, our study gave considerable insights into the status of food adulteration knowledge and practices in Lebanon.

## 5. Conclusions

The results of this study revealed that the knowledge and practices of identifying adulteration in the process of food purchase by consumers were lacking among most respondents. Increasing knowledge, awareness and motivation to identify food adulteration products during food shopping will empower consumers to improve buying practices, especially for the public with a lower level of education. Intensive public education campaigns to educate the entire populace to increase awareness levels and improve food safety knowledge to curtail the predominant use of food adulterants, and national regulatory agencies enacting relevant laws and regulations are also warranted.

## Figures and Tables

**Table 1:** Sociodemographic characteristics of study population (*n* = 499).

Characteristics	Study Sample, *n* (%)
Age (Mean ± SD)	26.4 ± 8.7
Gender	
Female	298 (59.7)
Male	201 (40.3)
Age range, years	
18–29	417 (83.6)
30–39	47 (9.4)
40–49	14 (2.8)
50+	21 (4.2)
Marital Status	
Single	415 (83.2)
Married	84 (16.8)
Area of Residency	
Beirut	142 (28.5)
Mount Lebanon	303 (60.7)
South	15 (3.0)
North	20 (4.0)
Beqaa	19 (3.8)
Education Level	
High School Diploma	74 (14.8)
Undergraduate (bachelor’s degree)	260 (52.1)
Master’s degree	147 (29.5)
PhD	18 (3.6)
Employment Status	
Employed (Full time)	215 (43.1)
Employed (Part time)	60 (12.0)
Actively seeking employment	39 (7.8)
Unemployed/Stay at home parent	39 (7.8)
Student	143 (28.7)
Retired	3 (0.6)
Total Income ^1^	
<LBP 1,000,000	46 (9.2)
LBP 1,000,000–3,000,000 LBP	141 (28.3)
LBP 3,000,000–5,000,000 LBP	102 (20.4)
>LBP 5,000,000	198 (39.7)

^1^ USD 1 = 6000 Lebanese pound. This average exchange rate was applicable at the time of the study as the dollar was fluctuating between LBP 5000–7000.

**Table 2:** Knowledge of food adulteration of the respondents.

Knowledge	Study Sample, *n* (%)
How food is adulterated *	
Rotten bread	355 (71.1)
Water added to a milk bottle	232 (46.5)
Chalk added to spices	194 (38.9)
Adding coloring dyes	239 (47.9)
Changing the expiry date	291 (58.3)
False claims	173 (34.7)
Common Adulterants *	
Urea	71 (14.2)
Illegal colorants and preserves	262 (52.5)
Pebbles	71 (14.2)
Chalk	160 (32.1)
Sand	97 (19.4)
Water	137 (27.5)
Food items that can be adulterated *	
Fruits and vegetables	133 (26.7)
Flour, wheat and bakery products	252 (50.5)
Chicken, meats and meat products	297 (59.5)
Juices	255 (51.1)
Milk	261 (52.3)
Spices	174 (34.9)
Salt and sugar	121 (24.2)
Rice	97 (19.4)
Can adulteration affect health?	
Yes	384 (77.0)
No	14 (2.8)
I do not know	101 (20.2)
Total Knowledge Score (Mean ± SD)	7.74 ± 4.49

* Multiple responses.

**Table 3:** Simple and multiple linear regression analyses for the association of characteristics of study participants with the knowledge score.

	Simple Linear Regression		Multiple Linear Regression	
Predictors	*β* Coefficient, (95% CI)	*p*-Value	*β* Coefficient, (95% CI)	*p*-Value
Gender	−0.900	0.031	−0.803	0.066
	[−1.718, 0.081]		[−1.660, 0.054]	
Age				
18–29 (ref.)	0			
30–39	1.368	0.048	0.460	0.574
	[0.012, 2.723]		[−1.145, 2.065]	
40–49	1.124	0.356	−0.157	0.911
	[−1.269, 3.518]		[−2.901, 2.588]	
50+	−0.161	0.872	−0.932	0.478
	[−2.131, 1.809]		[−3.510, 1.646]	
Marital Status	1.096	0.041	0.855	0.287
	[0.043, 2.148]		[−0.720, 2.430]	
Area of Residency				
Beirut (ref.)	0			
South	−2.197	0.071		
	[−4.584, 0.190]			
North	1.303	0.223		
	[−0.797, 3.402]			
Mount Lebanon	−0.676	0.138		
	[−1.570, 0.218]			
Beqaa	−0.776	0.478		
	[−2.924, 1.371]			
Education Level				
High School Diploma (ref.)	0			
Undergraduate (bachelor’s degree)	1.376	0.020	1.043	0.086
	[0.218, 2.534]		[−0.147, 2.234]	
Master’s degree	1.256	0.049	0.746	0.263
	[0.004, 2.509]		[−0.563, 2.054]	
PhD	−0.162	0.890	−0.533	0.656
	[−2.472, 2.147]		[−2.878, 1.813]	
Employment Status				
Employed (Full time) (ref.)	0			
Employed (Part time)	0.459	0.482	0.155	0.816
	[−0.824, 1.742]		[−1.154, 1.464]	
Actively seeking	−0.196	0.802	−0.178	0.820
	[−1.725, 1.334]		[−1.711, 1.355]	
Unemployed/Stay at home parent	0.445	0.568	0.054	0.947
	[−1.084, 1.975]		[−1.558, 1.667]	
Student	−1.061	0.028	−1.051	0.038
	[−2.009, −0.112]		[−2.044, −0.059]	
Retired	−2.991	0.251	−2.470	0.378
	[−8.100, 2.119]		[−7.972, 3.031]	
Total Income				
<LBP 1,000,000 (ref.)	0			
LBP 1,000,000–3,000,000	−0.418	0.552		
	[−1.797, 0.962]			
LBP 3,000,000–5,000,000	−0.302	0.683		
	[−1.756, 1.152]			
>LBP 5,000,000	−0.584	0.385		
	[−1.905, 0.736]			

**Table 4:** Attitudes toward food product labeling and packaging of study population.

Characteristics	Total Sample *n* = 499 (%)
Do you mainly buy/consume branded or unbranded products	
Branded	404 (81.0)
Unbranded	95 (19.0)
What unbranded products do you consume	
Honey	293 (58.7)
Olive oil	325 (65.1)
Rice	80 (16.0)
Molasses	147 (29.5)
I never buy unbranded	92 (18.4)
Do you trust the labels on the packages	
Yes, I trust them completely	194 (38.9)
I only trust labels on imported brands	183 (36.7)
I only trust labels on local brands	12 (2.4)
I do not trust the labels	110 (22.0)
Does Lebanon have a law against food adulteration	
Yes	165 (33.1)
No	334 (66.9)
Do you believe it is being followed (*n* = 165)	
Yes	12 (2.4)
No	151 (30.3)
N/A	2 (0.4)
The level of food adulteration in Lebanon	
Low	59 (11.8)
Moderate, can happen with foods of low cost	211 (42.3)
High	229 (45.9)

**Table 5:** Buying practices of the study population.

Characteristics	Total Sample *n* = 499 (%)
Who is responsible for buying the groceries at home	
Myself	166 (33.3)
Parents	298 (59.7)
Spouse	35 (7.0)
Do you read food labels before buying/consuming any product?	
Yes	311 (62.3)
No	188 (37.7)
What is your focus (*n* = 311) *	
Ingredients	208 (41.7)
Nutrition fact sheet	169 (33.9)
Just calories	76 (15.2)
Storage instructions	73 (14.6)
Addition of food additives	65 (13.0)
Nutrition claims	101 (20.2)
Health claims	60 (12.0)
Criteria for buying/consuming any food product *	
Expiry date	408 (81.8)
Price	382 (76.6)
Brand	344 (68.9)
Appearance of package	156 (31.3)
Local products	107 (21.4)
Imported products	55 (11.0)

* Multiple responses.

## Data Availability

The data presented in this study are available on request from the corresponding author. The data are not publicly available due to privacy.

## Supplementary Materials

The following supporting information can be downloaded at: https://www.mdpi.com/article/10.3390/foods11203178/s1. Supplementary S1: Consent form for the study participants; Supplementary S2: Invitation script for participation in the study; Supplementary S3: Complete survey for the study.
