# Supplementary material for: Knowledge, Attitudes and Practices of the Lebanese Community toward Food Adulteration
Source: Foods. 2022 Oct 12;11(20):3178. doi: 10.3390/foods11203178 (PMC9601908; doi:10.3390/foods11203178)
Supplement: Supplementary file 1 [file foods-11-03178-s001.zip › KAP_Foods_Supplementary S1_Clean.pdf]

Supplementary S1

CONSENT FORM

Dear Participant,

You are invited to participate in a research study entitled “Food adulteration Knowledge, Attitudes and Practices (KAPs) among Lebanese adults: a cross-sectional study.”

This study is conducted by Dr. Samer Kharroubi, Department of Nutrition and Food Sciences, American University of Beirut. The main objective of this study is to investigate the knowledge, attitudes and practices of the Lebanese population on food adulteration.
This message invites you to read the consent document and consider whether you want to be involved in the study.

And to note that:

- This is not an official message from AUB
- Participation is completely voluntary
- This study will include a sample of 720 participants who are at least 18 years old, who participate in shopping for the needs of their home, and are currently residing in Lebanon
- Participants will be recruited via invitations posted on social media
- Completing the online questionnaire will take around 5-10 minutes
- Only the data you provide in the questionnaire will be collected and analyzed.
- The survey is anonymous and there are no personal or identifying information.
- The research team does not have access to your name or contact details
- Data collected will be monitored and may be audited by the IRB while assuring confidentiality
- You may download the consent form if you wish to keep a copy

POTENTIAL BENEFITS TO SUBJECTS AND/OR TO SOCIETY

You will not receive any payment for participation in this study. Also, there will be no direct benefits to you.

However, the studying the knowledge, attitudes and practices of the Lebanese population will provide us with valuable insight on how well informed this population is.

POTENTIAL RISKS TO SUBJECTS AND/OR SOCIETY

The risks of the study are minimal and your participation in this survey does not involve any distress.

CONFIDENTIALITY

The collected data will remain confidential and anonymous. It will be stored on the PI's password protected computer, and only the research team would have access to it. Data will be monitored and may be audited by the IRB while assuring confidentiality.

We will be using the information collected from the surveys for our master's thesis project, which is a requirement for our degree at the Department of Nutrition and Food Sciences. Findings from this study will be used for research purposes only.

PARTICIPATION AND WITHDRAWAL

If you voluntarily consent to take part in this study, you can change your mind and withdraw at any time without consequences of any kind. Refusal or withdrawal from the study will involve no penalty or loss of benefits to which you are otherwise entitled. Also, your refusal to take part in the study will not affect your relationship with AUB.

QUESTIONS ABOUT THE STUDY

If you have any questions or concerns about the study, you can contact Dr. Samer Kharroubi at sk157@aub.edu.lb

CONCERNS OR QUESTIONS ABOUT YOUR RIGHTS

If you have concerns about the study or questions about your rights as a participant, you can contact the American University of Beirut (AUB) Social and Behavioral Institutional Review Board (IRB) at irb@aub.edu.lb or AUB extension: 5445.

ACCESS TO THE SURVEY

If after reading the consent document and having your questions answered, you voluntarily agree to take part in the study, you can access the survey by answering the questions below.
