# Supplementary material for: Knowledge, Attitudes and Practices of the Lebanese Community toward Food Adulteration
Source: Foods. 2022 Oct 12;11(20):3178. doi: 10.3390/foods11203178 (PMC9601908; doi:10.3390/foods11203178)
Supplement: Supplementary file 1 [file foods-11-03178-s001.zip › KAP_Foods_Supplementary S2_Clean.pdf]

Supplementary S2

INVITATION SCRIPT

Invitation to Participate in a Research Study

This notice is for an AUB-IRB Approved Research Study for Dr. Samer Kharroubi at AUB.
(Phone: (01) 350 000 Ext: 4541)

(Email: sk157@aub.edu.lb)

It is not an Official Message from AUB

I am inviting you to participate in a research study about "Knowledge, Attitudes and Practices of the Lebanese Community toward Food Adulteration".

The purpose of this study is to investigate the knowledge, attitudes and practices of the Lebanese population towards food adulteration as well as identify factors, demographic characteristics for example, associated with food adulteration, and to identify areas of improvement, raise awareness and provide recommendations that could be beneficial for policy decision-makers

You will be asked to complete a short survey/questionnaire with demographic information

You are invited because we are targeting people who are at least 18 years old and are currently residing in Lebanon

The estimated time to complete this survey is approximately 5 to 10 minutes

The research is conducted online and is hosted on AUB server

Please read the consent form and consider whether you want to be involved in the study

If you have any questions about this study, you may contact the investigator/research team (May Khanafer, 71576740, mmk71@mail.aub.edu)
