# Supplementary material for: Knowledge, Attitudes and Practices of the Lebanese Community toward Food Adulteration
Source: Foods. 2022 Oct 12;11(20):3178. doi: 10.3390/foods11203178 (PMC9601908; doi:10.3390/foods11203178)
Supplement: Supplementary file 1 [file foods-11-03178-s001.zip › KAP_Foods_Supplementary S3_Clean.pdf]

Supplementary S3

SURVEY

Knowledge, Attitudes and Practices of the Lebanese Community toward Food Adulteration

Principle Investigator: Dr. Samer Kharroubi

Student: May Khanafer

A) Socio - demographic

1. Gender
   a. Male
   b. Female

2. Age group
   a.20 - 29
   b.30 - 39
   c.40 - 49
   d. 60+

3. Marital Status
   a. Single
   b. Married
   c. Divorced
   d. Widowed

4. Area of Residency
   a. Beirut
   b. South
   c. North
   d. Mount Lebanon
   e. Bekaa

5. Highest level of education achieved
   a. High School Diploma
   b. Undergraduate degree (Bachelor Degree)
   c. Master’s Degree
   d. PhD
   e. Technical school

6. What is your current employment status?
   a. Employed full- time
   b. Employed part-time
   c. Seeking employment
   d. Unemployed/ Stay-at- home parent
   e. Student
   f. Retired

7. What is the total monthly income of your household?
   a. <500$

b. 500 - 1000$
c.1000 - 2000$
d. >2000$)

B) Buying Practices

1. Who buys the grocery?
    - Myself
    - Parents
    - Spouse

2. Do you read food labels before buying/ consuming any product? (Yes/No)

3. If yes, what do you focus on?
    - Ingredients
    - Nutrition Fact Sheet
    - Just Calories
    - Storage instructions
    - The addition of food additives
    - Nutrition claims (gluten free, lactose free, high in fiber…)
    - Health claims (heart healthy, calcium for healthy bones …)

4. What do you most look out for while buying/ consuming any product?
    - Expiry Date
    - Price
    - Brand
    - Local products
    - Imported products
    - Appearance of packaging

5. In your household, do you buy/ consume mainly branded or unbranded foods? Ex: Mazola olive oil (branded) or not?   (Branded/Unbranded)

6. If you buy/ consume unbranded, what are the products?

    - Honey
    - Olive oil
    - Rice
    - Molasses

- Other, please specify…

7. Do you trust the labels (ingredients, expiration dates, nutrition label etc…) placed on the packages?

- Yes, I trust them completely
- I only trust labels on imported brands
- I only trust labels on local brands
- I don’t trust the labels

C) Knowledge on Adulteration

1. How is food adulterated? Tick ✓or X next to the following options
   - Rotten bread
   - Water added to a milk bottle
   - Chalk added to turmeric powder
   - Coloring dyes added to tea
   - Changing the expiry date
   - Claiming milk is ‘lactose free’ although it is not

2. Do you believe that adulterating food may affect your health? (Yes or No)

3. Which substance(s) do you believe can be considered adulterants?

- Urea
- Coloring
- Pebbles
- Chalk
- Sand
- Water
- Don’t know

4. Have you ever experienced food adulteration from bought goods? (Yes/No)

5. If yes, what was your reaction? Tick ✓or X next to the following option(s)

- Directly threw away the food
- Call the company on their Customer Hotline and informed them
- Call the supermarket from where I bought the food and informed them
- Stopped purchasing this brand

- Gave the brand another chance
- Nothing

6. Which foods do you believe can be adulterated? Tick ✓ or X next to the following option(s)

- Fruits & Vegetables
- Flour, wheat and bakery products
- Meats and meat products
- Juices
- Honey
- Milk
- Spices
- Salt
- Sugar
- Rice
- All the above
- I don’t Know

7. Do you believe that Lebanon has a law against adulteration? (Yes / No)
8. If yes, do you believe it is being followed? (Yes / No)
9. What do you believe is true (choose 1); the level of food adulteration in Lebanon is:

- Low, hardly happens
- Moderate, can happen with foods of low cost
- High
